# Gratitude in Organizations: A Contribution for Healthy Organizational Contexts

**DOI:** 10.3389/fpsyg.2017.02025

**Published:** 2017-11-17

**Authors:** Annamaria Di Fabio, Letizia Palazzeschi, Ornella Bucci

**Affiliations:** Department of Education and Psychology (Psychology Section), University of Florence, Florence, Italy

**Keywords:** gratitude, organizations, positive psychology, primary prevention, well-being in the workplace, healthy organizations

## Abstract

This article reviews the construct of gratitude. Gratitude has been shown to be a fundamental resource for strengthening individual well-being. From a positive psychology perspective, gratitude is recognized as a promising opportunity for individuals because it can be enhanced through specific training according to a primary prevention framework. In organizations, gratitude is now thought to be crucial to employees’ efficiency, success, and productivity while also improving organizational citizenship behaviors, prosocial organizational behavior, and the organizational climate. Thus, gratitude is noteworthy because it increases positive relationships, social support, and workers’ well-being, reduces negative emotions at the workplace, and enhances organizational health and success. This perspective article concludes by suggesting new directions for gratitude research and intervention in the organizational context.

## Introduction

Although the word “gratitude,” or its linguistic equivalent, is present in almost every language, the concept has rarely been examined in the social sciences in general or organizational psychology in particular (Emmons and Crumpler, 2000). The construct of gratitude is traditionally a fundamental variable in the positive psychology framework (Seligman and Csikszentmihalyi, 2000; Seligman, 2002) and has been studied in relation to well-being (McCullough et al., 2002; Emmons and McCullough, 2003; Watkins et al., 2003, 2014; Wood et al., 2009, 2010; Lin, 2016). More recently, certain studies have focused on organizational contexts, analyzing not only the relationship between gratitude and well-being (Emmons, 2003) but also the relationship with other variables, in particular relational aspects such as positive relationships and social support at work (Hu and Kaplan, 2014), prosocial organizational behaviors (Michie, 2009; Grant and Gino, 2010), organizational citizenship behaviors (Dik et al., 2014), and teamwork and altruism (Dik et al., 2014). Gratitude also emerges as a variable of interest in relation to efficiency, success, productivity, and job performance (Emmons, 2003; Grant and Wrzesniewski, 2010). Gratitude could thus be considered as a promising means of promoting performance and healthy organizations. Therefore, gratitude is recognized as a significant resource for individuals (Emmons and Shelton, 2002; Snyder et al., 2014) and organizations (Fehr et al., 2017).

The importance of developing programs that enhance gratitude from a positive primary prevention perspective (Kenny and Hage, 2009; Di Fabio and Palazzeschi, 2015; Di Fabio and Kenny, 2016a,b) and a resources reinforcement perspective (Di Fabio, 2014, 2015; Di Fabio and Saklofske, 2014a,b) has also been highlighted.

## Defining Gratitude

The word “gratitude” originated from the Latin term *gratia*, which means grace, graciousness, or thankfulness (Emmons and Shelton, 2002). All words that derive from this Latin root “have to do with kindness, generousness, gifts, the beauty of giving and receiving, or getting something for nothing” (Pruysier, 1976, p. 69). Gratitude can be directed to human and non-human sources, such as God, nature, or animals (Weiner, 1986). It is an attribution-dependent state because it is the result of a cognitive process articulated in two stages. In the first stage, people recognize that they have achieved a positive result associated with feelings of happiness. In the second stage, people attribute their happiness to external sources, which creates a link between happiness and gratitude (Weiner, 1986). Ortony et al. (1988) theorize that gratitude is a combination of the admiration and joy experienced by individuals when they accept a gift from a benefactor. Lazarus (1991) defines gratitude as an empathic emotion. In fact, people are only able to experience gratitude when they recognize and appreciate what another individual did for them (Lazarus and Lazarus, 1994). McCullough et al. (2002) conceive of gratitude as an individual disposition, defining it as “a generalized tendency to recognize and respond with grateful emotion to the roles of other people’s benevolence in the positive experiences and outcomes that one obtains” (p. 112).

The multidimensionality of the construct emerged as the term evolved. Watkins et al. (2003) individuated three dimensions of gratitude: (1) a sense of abundance—grateful people do not experience feelings of deprivation in relation to their own life; (2) simple appreciation—grateful people can be characterized by their tendency to appreciate simple pleasures; and (3) appreciation of others—grateful people are able to appreciate the contribution of others to their well-being.

Subsequently, Adler and Fagley (2005) individuated eight dimensions of gratitude: (1) interpersonal—evaluation of the appreciation of others; (2) personal assets—focus on material and non-material goods; (3) present moment—concentration on here and now; (4) rituals of gratitude—to remind oneself of being grateful; (5) astonishment—frequency with which an individual remains enchanted; (6) social comparisons—the positive feelings that we experience when we compare our lives to that of others; (7) appreciation of life in general—from the awareness that it is not infinite; and (8) expression of gratitude—when people express their thankfulness.

## Gratitude and Positive Psychology

In the framework of positive psychology (Seligman and Csikszentmihalyi, 2000; Seligman, 2002), gratitude is conceived as appreciation of all the positive aspects of one’s own life (Emmons and Shelton, 2002; Wood et al., 2010). Gratitude was studied in relation to well-being and finding positive relationships (McCullough et al., 2002; Emmons and McCullough, 2003). Gratitude correlates positively with hope and optimism and negatively with depression, anxiety, and envy (McCullough et al., 2002). Watkins et al. (2003) demonstrated moderate to strong relationships between affective and cognitive aspects of well-being and gratitude. In a study by Froh et al. (2009), positive relationships were found between gratitude and positive affect, life satisfaction, optimism, and social support. Furthermore, gratitude is associated with well-being and is largely, though not completely, mediated by affect and belief (Toussaint and Friedman, 2009). Gratitude also predicts psychological well-being above the Big Five personality traits (Wood et al., 2009). A recent study (Lin, 2017) showed that higher-order gratitude, which consists of multiple components (i.e., thanking others, thanking God, cherishing blessings, appreciating hardship, and cherishing the moment), explained variance in integrated mental well-being, in terms of depression, self-esteem, and psychological well-being, after controlling for gender, age, religion, the Big Five personality traits, and unifactorial gratitude (GQ).

After considering the results present in the literature, trainings were developed to improve gratitude and, consequently, to promote well-being (Lyubomirsky et al., 2011; Rash et al., 2011). In a positive primary preventive perspective (Di Fabio and Saklofske, 2014a,b; Di Fabio and Kenny, 2016a), gratitude was considered a resource that should be enhanced early on in order to promote well-being.

## Gratitude in Organizations

Although the construct of gratitude in organizations has not been thoroughly studied, research emphasizes and confirms its fundamental role in organizational success (Emmons, 2003). Gratitude in organizations is crucial because it has a direct effect on improving the organizational climate and contributes to enhancing individual well-being and reducing negative emotions in the workplace, such as rancor and envy (Emmons, 2003). It is also important to employee efficiency, success, productivity, and loyalty (Emmons, 2003; Grant and Wrzesniewski, 2010). Gratitude thus appears to be a precious resource that sustains performance. Gratitude in working environments also promotes psychological safety at work (Edmondson, 2002): psychological security is considered the degree to which people perceive their work environment to be conducive to the expression of their own ideas, even if they could give a negative impression on of themselves. In other words, this offers individuals the security to take interpersonal risks. Workers who have a high level of psychological security believe that others will not penalize them for asking for help (Edmondson, 2002).

Part of the positive effect of gratitude on organizational well-being is related to it being an “antidote against toxic emotions at the workplace” (Emmons, 2003, p. 90), in particular against jealousy and the perception of injustice, both of which could have a negative effect on performance (Emmons, 2003). Gratitude positively influences attitudes toward others, for example the perception of support given by supervisors or colleagues, which in turn leads to increased satisfaction with interpersonal aspects of their workplace (Hu and Kaplan, 2014). Individuals who are grateful feel better, and their well-being helps them see their colleagues in a more positive light, thereby improving organizational citizenship behaviors and strengthening reciprocity, teamwork, and altruism (Dik et al., 2014). Gratitude also ensures that workers will be recognized for their organizational contributions (Dik et al., 2014).

A relationship has been established between gratitude and organizational citizenship behaviors (McCullough et al., 2001; Spence et al., 2013), which are tasks that workers carry out in organizations that help to maintain, support, and improve the organizational context. They are strictly connected to job performance (Organ, 1997). The association between gratitude and organizational citizenship behaviors underline as gratitude lead people to have an altruistic behavior because it has a function of moral incentive (McCullough et al., 2001; Spence et al., 2013). The function of gratitude as a moral incentive is explained by social exchange theory, through which organizational citizenship behaviors are understood (Konovsky and Pugh, 1994). From this perspective, individuals carry out organizational citizenship behaviors because they feel obliged to repay the positive treatment bestowed by the leaders and by the organization overall; otherwise, they would not realize such behaviors (Spence et al., 2013).

Gratitude is also associated with prosocial organizational behaviors (Michie, 2009; Grant and Gino, 2010) increasing well-being and performance (Cameron et al., 2004). Prosocial organizational behaviors consist of “helping, sharing, donating, cooperating, and volunteering.... [These] are positive social acts carried out to produce and maintain the well-being and integrity of others” (Brief and Motowidlo, 1986, p. 710). Prosocial behavior benefits the well-being of both workers and the organization as a whole (McNeely and Meglino, 1994). People who are thanked for their prosocial behavior are more likely to help again; moreover, they are more inclined to help others, thus creating a virtuous circle linked to the positive moral reinforcement of gratitude (Michie, 2009; Grant and Gino, 2010).

The contribution of gratitude to generalized reciprocity mechanisms has also emerged in organizational literature (Baker and Bulkley, 2014). Generalized reciprocity is based on the principle, “I help you, and you will help someone else,” and is recognized as a component of social capital in organizations. One way this occurs is through the act of reciprocating favors or passing favors to someone else. This behavior is based on positive emotions as it is aroused by the gratitude produced when an individual receives a favor from another person (Baker and Bulkley, 2014).

A recent approach by Müceldili et al. (2015), focused on a positive organizational perspective, examined the importance of gratitude at the collective level in organizations. They define collective gratitude as a positive emotional state shared within the group and being grateful for the good things that happen (Müceldili et al., 2015). Collective gratitude was found to enhance contextual performance, team learning, and high quality connections in organizations (Müceldili et al., 2015). Even more recently, Fehr et al. (2017) developed a multilevel model of gratitude in organizations that includes episodic gratitude at the event level, persistent gratitude at the individual level, and collective gratitude at the organizational level. Episodic gratitude is defined as “a feeling of appreciation in response to an experience that is beneficial to, but not attributable to, the self” (Fehr et al., 2017, p. 363). Persistent gratitude is considered “a stable tendency to feel grateful within a particular context” (Fehr et al., 2017, p. 363). Collective gratitude is “persistent gratitude that is shared by the members of an organization” (Fehr et al., 2017, p. 364). These different types of gratitude relate to various outcomes in organizations: episodic gratitude to organizational citizenship, persistent gratitude to well-being and communal exchange, and collective gratitude to organizational resilience and corporate social responsibility (Fehr et al., 2017).

According to this model, organizations interested in maintaining an optimal level of performance and organizational well-being should ask how they might cultivate and stimulate acts of gratitude in their members. Studies that address training methods to enhance gratitude highlight the benefits of training and suggest integrating it into leadership and management training programs (Shelton, 2000; Emmons, 2003; Fehr et al., 2017).

## Measuring Gratitude

The literature reveals different questionnaires to measure gratitude, which have changed as the construct has evolved over the last 15 years. The first questionnaire developed to measure gratitude is the Gratitude Questionnaire-6 (McCullough et al., 2002). This instrument considers gratitude as a generalized tendency to recognize and respond with grateful emotion to other people’s benevolence. It consists of six items, including “I am grateful to a wide variety of people” and “I have so much in life to be thankful for,” and is unidimensional. According to the same definition of the construct, McCullough et al. (2002) developed a short instrument, the Gratitude Adjective Checklist, composed of three adjectives (grateful, thankful, and appreciative).

Subsequently, questionnaires to measure gratitude as a multidimensional construct were developed. The Gratitude Resentment and Appreciation Test (Watkins et al., 2003) is comprised of 44 items and individuates three dimensions: sense of abundance (“Life has been good to me”), simple appreciation (“Often I’m just amazed at how beautiful the sunset is”), and appreciation of others (“I couldn’t have gotten where I am today without the help of many people”).

Another multidimensional questionnaire is the Appreciation Scale, which measures the eight dimensions outlined by Adler and Fagley (2005). It is composed of 57 items. The eight dimensions are interpersonal (“I remind myself to appreciate my family”); personal assets (“I remind myself to think about the good things I have in my life.”); present moment (“I stop and enjoy my life as it is”); rituals (“I stop to give thanks for my food before I eat); astonishment (“I have moments when I realize how fortunate I am to be alive”); social comparison (“I think of people who are less fortunate than I am to help me feel more satisfied with my circumstances”); appreciation of life in general in relation to loss and adversity (“Experiences of loss have taught me to value life”); and expression of gratitude (“I say “please” and “thank you” to indicate my appreciation”).

Spence et al. (2013) developed the State Gratitude Scale to evaluate the actual experience of gratitude, conceptualized as a transitory state that is discrete and episodic in nature. It is composed of five items, including “I feel grateful,” “I feel a warm sense of appreciation,” and “I have benefited from the goodwill of others.” However, this questionnaire considers the construct of gratitude to be unidimensional.

Two years later, Martini et al. (2015) created the Perceived Gratitude Scale, a nine-item instrument that specifically measures the perception of gratitude of users to socio-sanitary operators. The scale has a bi-dimensional structure that measures gratitude expressed by users’ (“Several users express gratitude for the care we offer them”) and gratitude as a source of support (“Some users’ gratitude compensates for the efforts you make at workplace”).

Due to the number of different questionnaires present in the literature, Morgan et al. (2017) created the Multi-Component Gratitude Measure (MCGM) to evaluate four components of gratitude: conceptions of gratitude, grateful emotions, attitudes toward gratitude (including motivational aspects and evaluations of its importance), and gratitude-related behaviors. Respondents are presented with scenarios to examine their understandings of gratitude.

## Conclusion

In a positive psychology framework (Seligman and Csikszentmihalyi, 2000; Seligman, 2002), gratitude is considered a fundamental individual resource (Emmons and Shelton, 2002; Snyder et al., 2014), and a promising individual strength in the organizational context (Fehr et al., 2017). In fact, gratitude seems crucial for employees’ efficiency, success, productivity, and well-being. Based on these promising findings, future research should continue to study the relationship between gratitude and efficiency, success, and productivity in different organizational contexts. Furthermore, future research on the relationship between gratitude and well-being in organizational contexts could examine the association between gratitude and hedonic well-being (Watson et al., 1988) and eudemonic well-being (Ryan and Deci, 2001; Waterman et al., 2010).

Gratitude appears to be essential to constructing positive relationships, a central feature for healthy organizations (Blustein, 2006, 2011; Di Fabio, 2016; Di Fabio and Kenny, 2016a; Di Fabio et al., 2016), and to developing new positive ways to conceptualize organizational relationality. While the connections between gratitude and prosocial organizational behaviors, organizational citizenship behaviors, and social support at the workplace have been underlined, it is also possible to analyze gratitude in relation to the new construct of workplace relational civility (Di Fabio and Gori, 2016a), which includes relational decency, relational culture, and relational readiness, and to the new construct of acceptance of change (Di Fabio and Gori, 2016b). A leadership style aimed at promoting gratitude at 360 degree could also be introduced (Avolio and Gardner, 2005; Michie, 2009). Under this perspective, the leader shows thankfulness to collaborators, while colleagues help collaborators to be grateful to both the leader and other employees, creating a cycle of gratitude that could lead to many positive effects in both performance and well-being for workers and for the entire organization. In this framework it is also possible to refer to positive organizational behavior (Luthans and Youssef, 2007; Youssef and Luthans, 2007; Avey et al., 2008; Bakker and Schaufeli, 2008), underlining the impact of a strategic management of human capital and of workers’ resources, among which gratitude could be one, for the improvement of organizational performance.

Regarding measures of gratitude in organizations, it should be noted that no questionnaires have been developed to detect gratitude in these specific contexts. Therefore, attempts should be made to create instruments to measure the constructs of episodic gratitude, persistent gratitude, and collective gratitude as defined by Fehr et al. (2017) specifically for organizational contexts.

It seems thus promising to develop gratitude to enhance positive relationships and new forms of organizational relationality (Di Fabio and Gori, 2016a) on the one side, on the other side to promote positive management and leadership styles based on gratitude (Avolio and Gardner, 2005; Michie, 2009). In this framework at an intervention level, gratitude is thus a particularly interesting construct because it can be enhanced through targeted training (Rash et al., 2011; Lai and O’Carroll, 2017). In a positive primary prevention perspective (Kenny and Hage, 2009; Di Fabio and Kenny, 2016a) for building strength (Di Fabio and Saklofske, 2014a,b; Di Fabio, 2015), it could be important to introduce early interventions aimed at improving gratitude in organizational contexts. From this perspective, it could even be seen as essential to pre-emptively intervene to enhance gratitude in organizations at different levels (individual, relational, organizational, inter-organizational) to promote performance, positive relationships, healthy workers, and healthy organizations.

## Author Contributions

ADF conceptualized the work and ideated the structure. ADF, LP, and OB analyzed the literature, and all authors wrote the manuscript. Then all authors read and revised the manuscript several times.

## Conflict of Interest Statement

The authors declare that the research was conducted in the absence of any commercial or financial relationships that could be construed as a potential conflict of interest.
